# Tumor PHD2 Expression Is Correlated With Clinical Features and Prognosis of Patients With HCC Receiving Liver Resection

**DOI:** 10.1097/MD.0000000000000179

**Published:** 2014-12-02

**Authors:** Li Zhen, Ning Shijie, Zhang Shuijun

**Affiliations:** From the Department of Colorectal and Anal Surgery (LZ); Department of Vascular Surgery (NS); and Department of Hepatobiliary Surgery (ZS), The First Affiliated Hospital of Zhengzhou University, Zhengzhou, China.

## Abstract

The role of prolyl hydroxylase domain protein 2 (PHD2) in carcinogenesis has been studied in a variety of cancer types. However, the association between PHD2 and human hepatocellular carcinoma (HCC) has not been documented.

A total of 220 patients with primary HCC who underwent a curative liver resection were enrolled in this study. The tumor samples were obtained during the surgical procedure from each patient for PHD2 immunohistological staining. All the patients were followed up and the disease-free survival (DFS) and overall survival (OS) were evaluated.

We found that that high PHD2 expression was significantly associated with higher stage (stages III + IV) (odds ratio [OR] = 5.576, *P* < 0.001), larger tumor size (>5 cm) (OR = 6.176, *P* < 0.001), poorer tumor differentiation (OR = 1.424, *P* = 0.003), and higher serum alpha fetoprotein (AFP) level (OR = 6.861, *P* < 0.001). Compared to those with high PHD2 expressions, patients with low PHD2 expression had significantly longer DFS and OS periods (both *P* < 0.001). Cox regression analyses revealed that higher levels of PHD2, tumor size, tumor stage, as well as serum AFP level were predictors for a worse prognosis in patients with HCC.

PHD2 expression in the tumors is associated with the clinical features and prognosis of patients with HCC; it may be used as a histological marker for HCC.

## INTRODUCTION

Hepatocellular carcinoma (HCC) is one of the most common cancers worldwide with a low 5-year postoperative survival rates.^1,2^ To date, the molecular mechanism of development and progression of HCC remains largely unknown. Early diagnosis is critical to improve the prognosis of HCC. Serum alpha fetoprotein (AFP) has been the most widely used tumor marker for HCC. However, its sensitivity and specificity remain controversial.

Hypoxia is a feature of many tumors including HCC, and the ability of tumor cells to adapt to reduced oxygen and nutrient supply is vital for their survival.^3^ Hypoxia-inducible factor-1 (HIF-1) is a key regulator of cellular response to hypoxia and has been suggested as playing an important role in the regulation of tumor cell proliferation, survival, migration, and metastasis. Stability of the HIF-1α subunits is regulated by HIF prolyl 4-hydroxylases (PHD1–3). The associations between PHD family members (PHD1–3) have been reported. PHD1 overexpression inhibits tumor growth and neovascularization in colon carcinoma cells via suppressing HIF-1α accumulation.^4^ PHD1 also blocks nuclear factor-κB-mediated cyclin D1 expression and proliferation in lung carcinoma cells.^3^ PHD3 methylation level increases in breast cancer and leukaemic cell lines.^5^ High PHD3 expression is significantly correlated with poor overall survival in pancreatobiliary ampullary adenocarcinoma.^6^ In nonsmall cell lung cancer (NSCLC), the high expression of PHD3 was correlated with an early tumor stage and better differentiation.^7^ Another study showed that overexpression of the PHD1–3 are individually and collectively unfavorable prognosticators for NSCLC survival.^8^

PHD2 is a major oxygen/redox-sensitive enzyme that induces cellular adaptation to stress conditions via regulating the activation of HIF1 in cells.^9^ Knockdown of PHD2 inhibits tumor growth of human breast cancer cells by affecting transforming growth factor-β.^10^ PHD2 inhibition diminishes tumor growth through matrix metalloproteinase-induced transforming growth factor-β activation.^11^ PHD2 expression is associated with the tumor grade and poor prognosis in patients with colorectal cancer^12^ and pancreatic endocrine tumors.^13^ Loss of PHD2 in myeloid cells and T-lymphocytes impairs tumor development.^14^ PHD2 is also indicated as a strong prognostic marker in human gastric cancer.^15^

A recent study in mice showed that inhibition of PHD2 increases the HCC occurrence and stimulates the development of cholangiocarcinoma.^16^ However, little is known about the association between PHD2 and HCC in a clinical setting. In addition, the effect of PHD2 on the biological behaviors of HCC cells remains largely unknown.

## METHODS

### Subjects and Specimen Collection

A total of 220 patients with primary HCC who underwent a curative liver resection were included in our hospital between January 2006 and December 2010. HCC samples were obtained during the surgical procedure from each patient. None of these patients underwent the preoperative chemotherapy/radiotherapy or postoperative adjuvant therapy. Twenty pairs of HCC biopsies with matched adjacent noncancerous normal liver tissues were frozen and stored in liquid nitrogen until further use. HCC diagnosis was based on the World Health Organization criteria.^17^ Tumor differentiation was defined according to the Edmondson grading system. Tumor staging was determined according to the sixth edition of the tumor-node-metastasis classification of the International Union against Cancer. The median follow-up time was 36.4 months (1–60 months). Disease-free survival (DFS) was calculated from the day of surgery to either relapse or death without relapse, and it was censored only for patients who were alive and recurrence free at the last follow-up. Overall survival (OS) was measured from the date of hepatectomy to the time of death or the last follow-up. The study protocol was approved by the ethics committee of Zhengzhou University. Written informed consent was obtained from each participant before data collection.

### PHD2 Immunohistological Staining

Formalin-fixed and paraffin-embedded 5-μm-thick tumor sections were deparaffinized, placed in a solution of absolute methanol and 0.3% hydrogen peroxide for 30 minutes, and treated with blocking serum for 20 minutes. The slides were incubated overnight with PHD2 antibody (Millipore Corporation, Billerica, MA) at a 1:150 dilution at 4°. The immune reaction was revealed with 0.06 mmol/L diaminobenzidine (DAB-Dako, DakoCytomation, Carpinteria, CA) and 2 mmol/L hydrogen peroxide. Sample scoring was performed by semiquantitative microscopic analysis, measuring the number of stained cells and signal intensity. Three spots were evaluated for each sample and a mean score was calculated. Based on the percentage of anti-PHD2 immune-positive tumor cells, a score of 1 was given when ≤10% of cells were positive; 2 when 10% to 50% of cells were positive; and 3 when ≥50% of cells were positive. Signal intensity was scored as negative (0), weak (1), moderate (2),^18^ and strong (3). Both scores were multiplied and the resulting score was used to categorize PHD2 expression as low (0–6) and high (>6) expressions.^19^

### Cell Culture

Three HCC cell lines (QGY7703, Bel7404, and Hep3B)^20^ and 1 normal hepatic cell line (Lo2) were cultured in Dulbecco modified Eagle medium (Invitrogen, Carlsbad, CA) supplemented with 10% fetal bovine serum (FBS; HyClone, Logan, UT) and 1% penicillin-streptomycin (Invitrogen, Grand Island, NY) at 37°C with 5% CO_2_.

### Silencing of PHD2 by Short-Interfering RNA

The QGY7703, Bel7404, and Hep3B cell lines were selected to conduct short-interfering RNA inhibition assay. Cells were 75% confluent and transfected with 5.0 nM PHD2 short-interfering RNA (siRNA) for 48 hours using Oligofectamine (Invitrogen GmbH, Darmstadt, Germany) according to the manufacturer's instructions. The negative control siRNA (Qiagen GmbH, Hilden, Germany) was used as negative control for all experiments. Sequences of PHD2 siRNA (Genbank accession number NM_022051) are corresponded to nucleotides 3901 through 3921 (aaggacatccgaggcgataag) and nucleotides 4077 through 4097 (aacgggttatgtacgtcatgt).^19^

### Western Blot

The cells were lysed for Western blot assay to determine PHD2 expression. Cell extracts were resolved on sodium dodecyl sulfate (SDS)-polyacrylamide gels followed by transfer to nitrocellulose membranes. Proteins were resolved by electrophoresis on 8% to 12% SDS–polyacrylamide gels and transferred by electroblotting to polyvinylidene difluoride membranes in transfer buffer (25 mM Tris-HCl (pH 7.6), 192 mM glycine, 20% methanol, 0.03% SDS). After immunoblot analysis, membranes were immunoblotted with anti-PHD2 antibody (1:1000; Santa Cruz Biotechnology, Santa Cruz, CA) and glyceraldehyde-3-phosphate dehydrogenase (1:1000; Santa Cruz Biotechnology). The membrane was incubated with the secondary antibody against rabbit immunoglobulin G. It was then examined with the enhanced chemiluminescence detection system (Amersham Bioscience Europe, Freiberg, Germany) according to the manufacturer's instructions.

### Cell Proliferation Assay

To evaluate proliferation rate, cells were seeded at 1.0 × 10^3^ per well in a 96-well plate in complete medium. After 3 days, MTT assay was performed according to manufacturer instructions (Sigma Aldrich, St. Louis, MO). Briefly, cells were washed with phosphate buffer saline. MTT reagent (3-(4, 5-dimethylthiazol-2-yl)-2, 5-diphenyltetrazolium bromide, M 2128, Sigma Aldrich) was added and incubated at 37°C for 3 hours. After MTT reagent removal, MTT solvent (10 % Triton × 100 and 0.1 N HCl in anhydrous isopropanol) was added. Absorbance was determined in a Microwell plate reader (Model 680; Biorad, Marnes-La-Coquette, France). We used 10 to 20 replicates for each cell line, and the experiment was repeated 3 times independently.

### Migration and Invasion Ability

Cell migration was measured using Corning transwell inserts (BD Biosciences, San Jose, CA) with 8.0 μm pore polycarbonate membrane. We used 1% FBS in the lower wells as chemoattractant. Cells were isolated with 0.25% trypsin digestion; 2 × 10^5^ cells were added to the top transwell chamber and cells were allowed to migrate for 24 hours. The nonmigrated cells were then removed from the upper chamber by using a cotton swab. Migrated cells washed with phosphate-buffered saline (PBS) and labeled with Calcein-AM (Abcam, Cambridge, MA). The nucleus was counterstained with 4’,6-diamidino-2-phenylindole (DAPI). Migrated cells were quantified in 3 to 5 fields/well with 2–3 wells/condition.

For invasion assays, the 8 μm pore HTS Fluoroblok filters (Becton Dickenson,Franklin Lakes, NJ) were coated with 300 μL Matrigel (100 μg/mL) overnight at 4°C. The matrix was rehydrated for 2 hours with serum-free media prior to use, then washed once with PBS; 2 × 10^5^ cells were seeded in the chambers at 37°C. After 48 hours, the cells invading to the bottom of the membrane were labeled with Calcein-AM and counted as described in migration assay.

## TERMINAL DEOXYNUCLEOTIDYL TRANSFERASE dUTP NICK END LABELING (TUNEL) ASSAY

The apoptosis in the cultured cell lines were detected by in situ DeadEndTM Colorimetric Apoptosis Detection System (Promega, Madison, WI, USA) according to the manufacturer's instructions. Briefly, cells were incubated with terminal deoxynucleotidyl transferase enzyme in a humidified chamber at 37°C for 60 minutes. The reaction was terminated by transferring the slides to 2× sodium citrate saline solution. The sections were counterstained with DAPI. For quantitative analyses, 5 fields per section were selected. Apoptosis was indexed by counting TUNEL-positive cells per 100 nuclei per section.

### Statistical Analyses

The χ^2^ test was used to analyze the relationship between the PHD2 expression and clinicopathologic features of patients with HCC. The odds ratios (ORs) and 95% confidence intervals (CIs) were calculated. Survival curves were obtained by the Kaplan–Meier method, and the statistical significance of the differences in survival among subgroups was evaluated with the log-rank test. The Cox proportional hazards model was employed to assess the independent prognostic values of PHD2 expression pattern. The hazard ratio and its 95% CI were calculated. Statistical analyses were all performed with SPSS software package (version 16.0; SPSS, Inc, Chicago, IL). All statistical tests were 2 sided, and *P* < 0.05 was considered to be statistically significant.

## RESULTS

**FIGURE 1:**
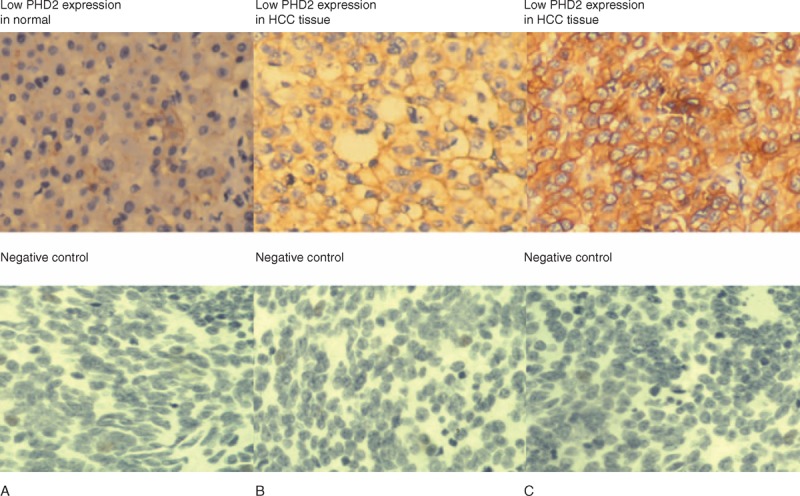
(A) PHD2 expressions in normal liver tissues and HCC samples by immunohistological staining assays. (B and C) Normal liver tissues show a weak PHD2 expression, while the PHD2 expression is higher in tumor samples. PHD2 = prolyl hydroxylase domain protein.

To compare the PHD2 expressions in normal liver tissues and HCC samples, we collected 20 pairs of HCC biopsy samples with matched adjacent noncancerous normal liver tissues (Figure 1). Compared to the normal liver samples, the PHD2 high expression was significantly prevalent in HCC samples (Table 1).

**TABLE 1:**
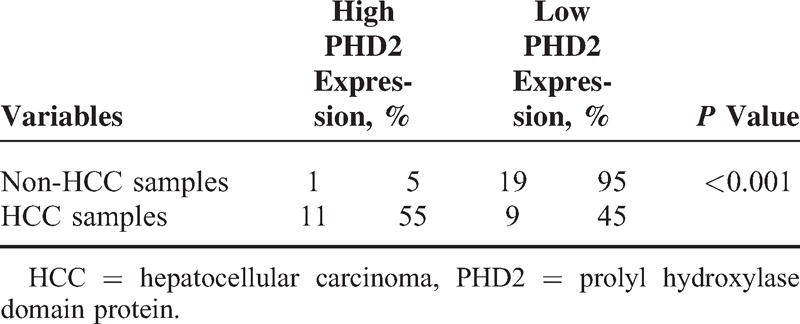
PHD2 Expressions in Noncancerous Normal Liver Tissues and HCC Sample

We next analyzed the association between the PHD2 expression in tumor pattern and the clinicopathological features of the HCC subjects. Table 2 shows that the high PHD2 expression was significantly associated with higher stage (stages III + IV) (OR = 5.576, *P* < 0.001), bigger tumor size (>5 cm) (OR = 6.176, *P* < 0.001), poorer differentiation (OR = 1.424, *P* = 0.003), and higher serum AFP level (OR = 6.861, *P* < 0.001). In contrast, the tumor PHD2 expression status was not associated with age, sex, cirrhosis, or lymph node metastasis in patients with HCC (all *P* > 0.05).

**TABLE 2:**
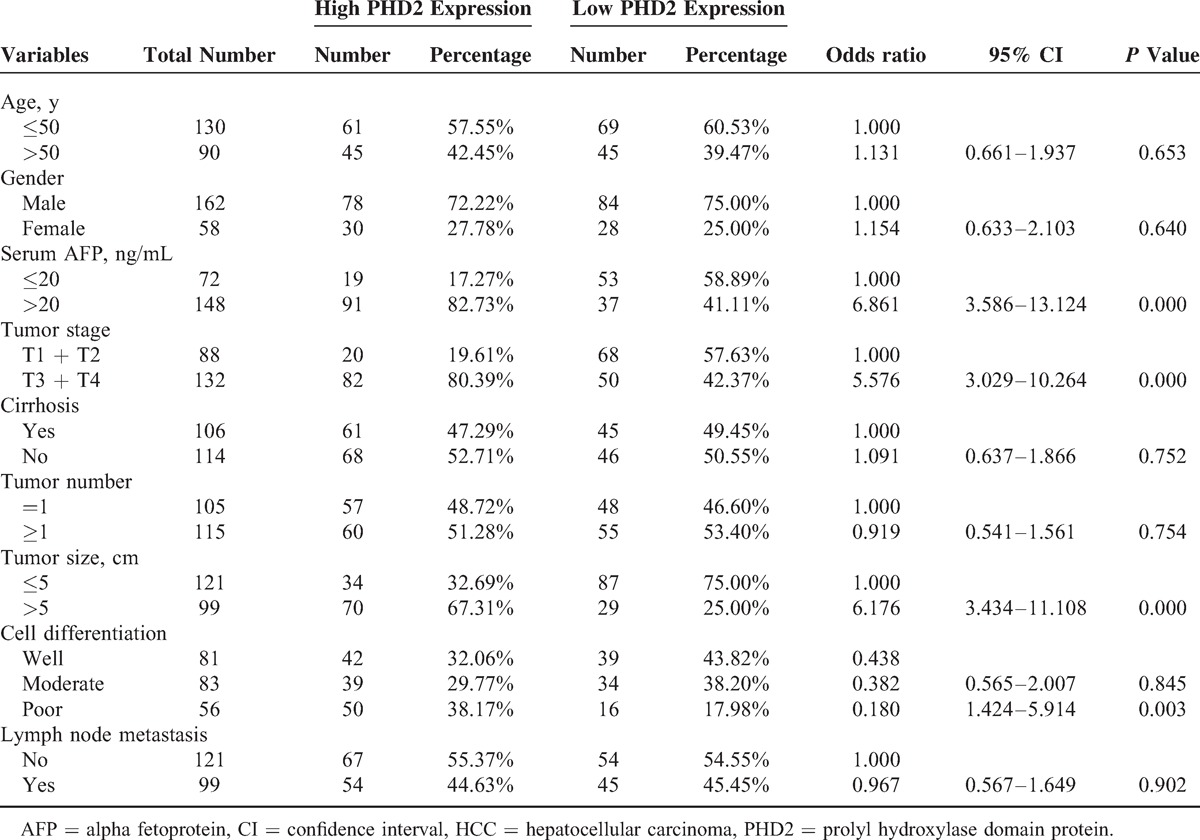
Association Between the PHD2 Expression and the Clinicopathological Features of the HCC Subjects

### High PHD2 Expression Is Associated With Poor Prognosis of Patients With HCC

**FIGURE 2:**
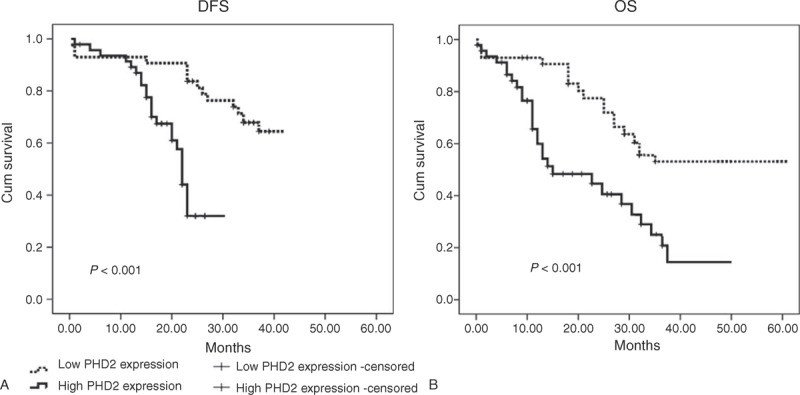
Kaplan–Meier curves in patients with HCC according to their PHD2 expression status. Compared with patients with HCC having high PHD2 expression, those with low PHD2 expression had a longer (A) DFS period (25.5 ± 3.6 vs 16.7 ± 3.4 months, log-rank, *P* < 0.001). (B) Patients with low PHD2 expression had longer OS times, whereas those with high PHD2 expression had shorter survival times (37.5 ± 3.6 vs 29.5 ± 4.7 months, log-rank, *P* < 0.001). DFS = disease-free survival, HCC = hepatocellular carcinoma, OS = overall survival, PHD2 = prolyl hydroxylase domain protein.

The log-rank test shows that the DFS and OS periods were significantly different between patients with low and high PHD2 expressions (Figure 2). Compared with patients with HCC having high PHD2 expression, those with low PHD2 expression had a longer DFS period (25.5 ± 3.6 vs 16.7 ± 3.4 months, log-rank, *P* < 0.001, Figure 2A). Patients with low PHD2 expression had longer OS times, whereas those with high PHD2 expression had shorter survival times (37.5 ± 3.6 vs 29.5 ± 4.7 months, log-rank, *P* < 0.001, Figure 2B). Univariate Cox regression analyses revealed that higher levels of PHD2, tumor size, tumor stage, as well as serum AFP levels were all worse predictors for HCC prognosis. The multivariate Cox regression analysis further confirmed that the abovementioned variables were independent prognostic markers for DFS and OS of patients with HCC (Table 3).

**TABLE 3:**
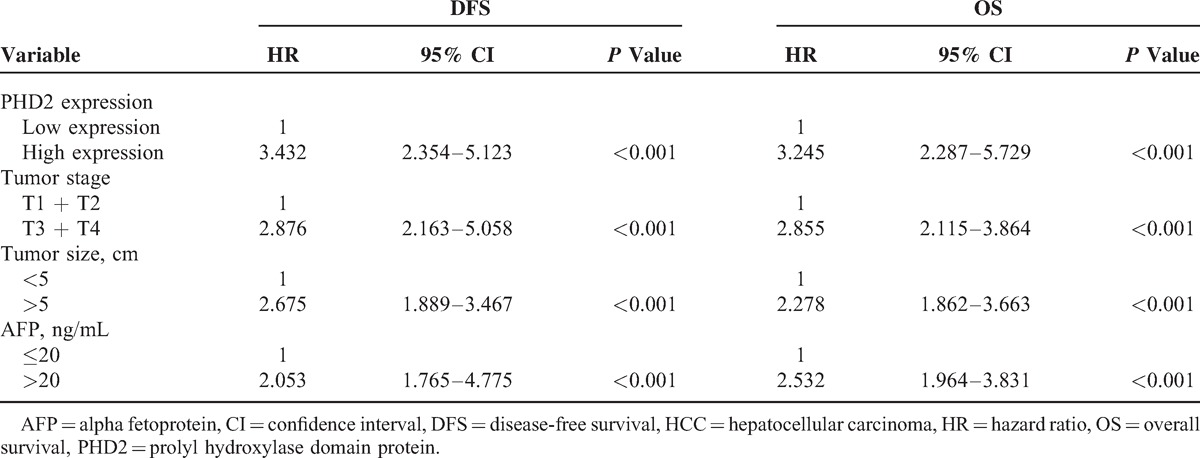
Multivariate Cox Proportional Regression Analysis on PFS and OS of Patients With HCC

### PHD2 Regulates the Proliferation, Migration, Invasion and apoptosis of HCC Cell Lines

**FIGURE 3:**
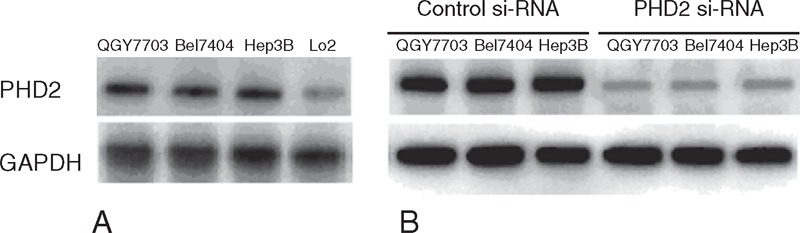
PHD2 protein expressions in HCC cell lines (QGY7703, Bel7404, and Hep3B) by Western blot assays. (A) Our data show that the HCC cell lines had significantly higher PHD2 expressions than normal hepatic cell line (Lo2). (B) After si-RNA transfection, the PHD2 protein in HCC cell lines was significantly inhibited. HCC = hepatocellular carcinoma, PHD2 = prolyl hydroxylase domain protein.

**FIGURE 4:**
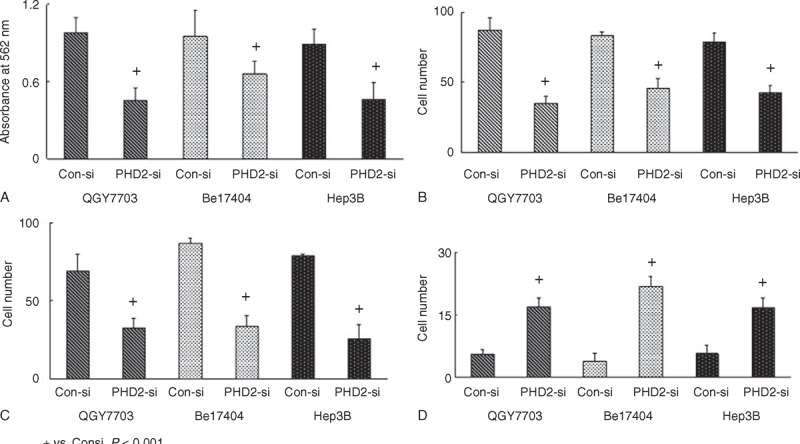
Biological behaviors of HCC cells after PHD2 inhibition. (A) Cell growth rate significantly inhibited in HCC cell lines after PHD2 si-RNA transfection by MTT assay. (B) Cell migration abilities significantly decreased after PHD2 silencing. (C) PHD2 gene silencing dramatically inhibited the invasion abilities of these HCC cells. (D) PHD2 inhibition markedly increased cell apoptosis in cultured HCC cell lines compared to control si-RNA transfection. HCC = hepatocellular carcinoma, PHD2 = prolyl hydroxylase domain protein.

**FIGURE 5:**
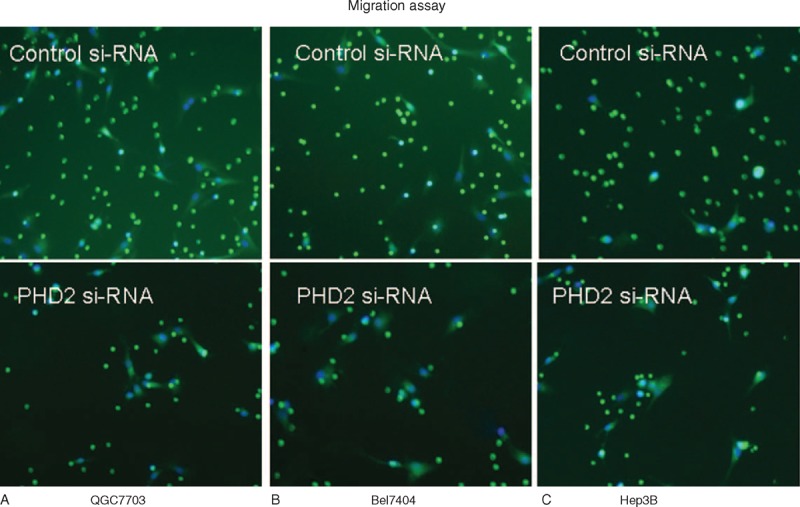
Representative images of migration assay using transwell (magnification: ×100). Cells were added to the top transwell chamber and allowed to migrate for 24 hours. Our data show that the PHD2 inhibition in (A) QGY7703, (B) Bel7404, and (C) Hep3B considerably reduced their migration abilities. Migrated cells were labeled with Calcein-AM. The nucleus was counterstained with DAPI. Migrated cells were quantified in 3–5 fields/well with 2–3 wells/condition. DAPI = 4’,6-diamidino-2-phenylindole, PHD2 = prolyl hydroxylase domain protein.

**FIGURE 6:**
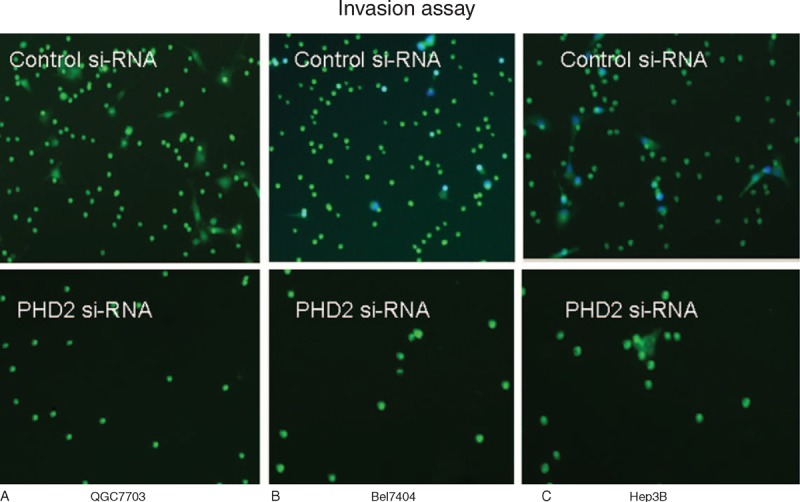
Representative images of stained cells on the underside of the insert in invasion assay (magnification: ×100). Our data show that the PHD2 inhibition in (A) QGY7703, (B) Bel7404, and (c) Hep3B considerably reduced their invasive abilities. Invading cells were labeled with Calcein-AM and quantified in 3 to 5 fields/well with 2 to 3 wells/condition. PHD2 = prolyl hydroxylase domain protein.

Western blot results showed that the PHD2 protein expressions were significantly higher in HCC cell lines (QGY7703, Bel7404, and Hep3B) than normal hepatic cell line (Lo2) (Figure 3A). Western blot results showed that the PHD2 protein expressions in HCC cell lines were significantly inhibited by the PHD2 siRNA silencing technique (Figure 3B). The cell proliferation assays revealed the cell growth rate was significantly inhibited in abovementioned HCC cell lines after PHD2 siRNA transfection compared with cells treated with the control siRNA (Figure 4A). Cell migration assay showed that PHD2 knockdown significantly decreased the numbers of migrated cells (Figure 4B). Furthermore, silencing of the PHD2 gene dramatically inhibited the invasion of the QGY7703, Bel7404, and Hep3B cells by number (Figure 4C). Last, PHD2 knockdown induced a significantly increased cell apoptosis number in cultured HCC cell lines compared to control siRNA transfection (Figure 4D). The representative images of migration assay and invasive assay are shown in Figures 5 and 6.

## DISCUSSION

This study intends to unveil the association between PHD2 and outcomes of patients with HCC, and provide a potential new therapeutic target in treating HCC. Our data show that higher PHD2 expression in tumor is correlated to tumor stage, tumor diameter, and serum AFP levels in patients with HCC. More importantly, higher PHD2 expression is closely associated with a poorer prognosis of the patients with HCC who received surgical treatment.

HCC ranks fifth among the solid tumors worldwide and is most prevalent in Asia because of hepatitis B or C.^21^ Effective treatment for HCC, however, is currently limited, especially when patients present with symptoms. The 5-year survival for patients with HCC having cirrhosis ranges from 30% to 50%.^22^ Currently, systemic chemotherapy to patients with HCC who frequently have the comorbidity of cirrhosis is not effective because of relative poor chemosensitivity of HCC and the impairment of patients’ liver function resulting in the significant increases of the toxicity following chemotherapy.^23^ Identifying an alternative treatment method that is more effective but less toxic, thereby, is critical to improve the survival for patients with HCC.^24–26^

The development of HCC is multifactorial and may be involved in both genetic and epigenetic changes.^1^ From a pathophysiological point of view, oxidative stress from chronic liver diseases may be one of the most critical elements in the development of HCC.^1,23,27^ The PHD isoforms (PHD1–3) are oxygen-sensing molecules that are strictly regulated by the levels of oxygen tension (pO_2_).^28^ PHDs hydroxylate the α subunit of HIF-1 and HIF-2 in the oxygen-dependent degradation domain.^29,30^ Of 3 PHD isoforms in human, PHD2 is the key limiting enzyme that hydroxylates HIF-1α under normoxic conditions.^24,29,31^

PHD2 is a promising target for the treatment of HCC on several levels. First, when pO_2_ decreases, PHDs become less active resulting in HIF stabilization and, in turn, the initiation of adaptive responses, which include scavenging of reactive oxygen species, angiogenesis, erythropoiesis, and metabolic reprogramming,^24,32^ and, therefore, improve host's ability to protect against tumor cells. Second, reduced activity of PHD2 in healthy organ amplifies the antioxidative response and reduces the tissue damage from oxidative burst, which is considered as a key component in carcinogenesis of HCC, especially with recurrent liver cell necrosis and regeneration following damage due to virus and toxic agents.^1,24,27,33^ In addition, a recent study by Leite de Oliveira et al^24^ has showed that heterozygous deletion of PHD2 in endothelial cells does not affect the vessel density or lumen size but, rather, normalizes the endothelial lining, barrier, stability, and pericyte coverage, restores tumor oxygenation, and, therefore, inhibits metastasis of the tumor cells.^24,31,34^ Last, deletion of 1 or 2 PHD2 alleles in stromal cells improves tumor vessel perfusion and provides a better access for chemotherapy agents, such as doxorubicin, to the tumor site, without requiring direct deletion of PHD2 in the tumor cells.^35^

In consistent with those previous findings, we found that the stages, tumor sizes, and cancer-related survival are all inversely associated with the expression level of PHD2 in this cohort of patients with HCC. From this study, we also observed that the patients who were positively detected for the AFP intend to have a significantly higher level of PHD2, although it is unclear if there is a linear association and the involved mechanism. One may assume that the high expression of PHD2 is related to the advancement of the HCC, which results in the high production of AFP.

Interestingly, both tumor-promoting and suppressive effects of PHD2 have been previously described, although its unambiguous roles in tumorigenesis have been supported by numerous studies in pancreatic endocrine tumor, pheochromocytomas, and colorectal cancers.^24,25,26,36^ For example, overexpression of PHD2 was reported in the more aggressive phenotypes of squamous cell carcinoma in the head and neck.^29^ This was also correlated to the higher aggressiveness and recurrence rates in pancreatic endocrine tumors.^37^ On the other hand, increased PHD2 expression was associated with a better prognosis for patients with pancreatic cancer due to suppression of angiogenic cytokines, vascular endothelial growth factor, angiopoietin-1, and interleukin 8 under hypoxic conditions.^28^ One study, however, indicated a simultaneously increased level of HIF-1 and PHD2 in the head and neck squamous cell carcinomas, given PHDs hydroxylate HIFs at the prolyl residues.^29^ This paradoxical increased expression of PHD2 may be induced by the hypoxic condition during tumor aggression and forms a part of a feedback loop to limit hypoxic signal.^28^ From others, elevated PHD2 may not be sufficient to downregulate the expression level of HIF-1α in cancer frequently presenting with abnormal hypoxic signals.^34,38–40^ Etiology of HCC involved in the PHD2 pathway, therefore, is highly complicated. Among all, studying crosstalk between the PHD isoforms and the HIF members may be exceptionally important to understand the correlations between PHD2 and the development of HCC.

In our in vitro study, we observed that knockdown of PHD2 inhibits cell migration and invasion. However, the PHD2 does not significantly associate with tumor metastasis in our clinical study. The HCC metastasis is a very complex process in vivo. The PHD2 may be one factor associated with metastasis, but there are still many other regulating factors. To better clarify this discrepancy between the results in vitro and in vivo, an in vivo study of PHD2 knockdown or overexpression may be needed.

To our knowledge, this is the first study to outline the association between PHD2 and HCC in a clinical stetting. Studying the roles of PHD2 in the treatment of HCC is promising because of well-characterized hypervascular arterial feeding to HCC tumor cells.^23^ Our study provides evidence to suppress the expression of PHD2 in improving the survival of the patients with HCC. A repeat study with a larger number of patients is currently underway to validate this finding.
